# COVID-19 and Heart: From Clinical Features to Pharmacological Implications

**DOI:** 10.3390/jcm9061944

**Published:** 2020-06-22

**Authors:** Vincenzo Russo, Roberta Bottino, Andreina Carbone, Anna Rago, Andrea Antonio Papa, Paolo Golino, Gerardo Nigro

**Affiliations:** Division of Cardiology, Department of Translational Medical Sciences, University of Campania “Luigi Vanvitelli”, Monaldi Hospital, 80131 Naples, Italy; ro-bottino@hotmail.com (R.B.); andr.carbone@gmail.com (A.C.); anna_rago@alice.it (A.R.); andreantoniopapa@libero.it (A.A.P.); paolo.golino@unicampania.it (P.G.); gerardo.nigro@unicampania.it (G.N.)

**Keywords:** coronavirus disease 2019 (COVID-19), angiotensin-converting enzyme inhibitor, myocardial injury, cardiovascular diseases, cardiac biomarkers, arrhythmias

## Abstract

A highly pathogenic human coronavirus, severe acute respiratory syndrome coronavirus 2 (SARS-CoV-2), has been recently recognized in Wuhan, China, as the cause of the coronavirus disease 2019 (COVID-19) outbreak which has spread rapidly from China to other countries in the world, causing a pandemic with alarming morbidity and mortality. The emerging epidemiological data about COVID-19 patients suggest an association between cardiovascular diseases (CVD) and SARS-CoV-2 infection, in term of clinical features at hospital admission and prognosis for disease severity. The aim of our review is to describe the cardiological features of COVID-19 patients at admission, the acute cardiac presentation, the clinical outcome for patients with underlying CVD and the pharmacological implications for disease management.

## 1. Introduction

Human coronaviruses (HCoVs) are enveloped non-segmented positive-strand RNA viruses, with rapid evolution owing to its high genomic nucleotide substitution rates and recombination. HCoVs are associated with multiple respiratory diseases of varying severity, including common cold, pneumonia and bronchiolitis. A highly pathogenic HCoV, firstly identified as 2019-nCOV and now named severe acute respiratory syndrome coronavirus 2 (SARS-CoV-2), has been recently recognized in Wuhan, China, as the cause of the coronavirus disease 2019 (COVID-19) outbreak, with alarming morbidity and mortality. The clinical course of about 15% of COVID-19 patients may be complicated by the onset of a severe interstitial pneumonia, which may then progress towards acute respiratory distress syndrome (ARDS) and/or multi organ failure (MOF) and death. There are many emerging data on patients’ clinical features at hospital admission and on the factors influencing the prognosis in COVID-19 patients. The aim of our review is to describe the cardiological features of COVID-19 patients at admission, the acute cardiac presentation, the clinical outcome for patients with underlying cardiovascular diseases (CVD), the pharmacological implications for disease management.

## 2. Pathogenetic Mechanisms

SARS-CoV-2 has been classified by the World Health Organization (WHO) as a beta coronavirus of group 2B for its acid sequence similarity with SARS-CoV and middle east respiratory syndrome (MERS)-CoV, the most pathogenic known beta coronaviruses [1,2]. SARS-CoV-2, as typical of coronaviruses, encodes at least 27 proteins, including 15 non-structural proteins, four structural proteins, and eight auxiliary proteins [3]. Among the structural proteins, spike glycoprotein S (S) is located on the outer envelope of the virion and mediates surface entry of coronaviruses into host cells. Its biochemical composition consists of a large ectodomain, a single-pass transmembrane anchor, and a short C-terminal intracellular tail [4]. S1 receptor binding unit and S2 membrane fusion unit are part of the coronaviruses ectodomain of S protein. The receptor binding domain (RBD) of the S1 unit determines the capability to enter host cells by binding a high affinity receptor while S2 unites allowing membrane fusion [4]. The S1 RBD of SARS-CoV-2 S glycoprotein was found to have almost identical 3-D structure and a high degree of homology with the RBD of SARS-CoV [4,5,6]. Previous studies have demonstrated that SARS-CoV RBD has a strong binding affinity to angiotensin-converting enzyme 2 (ACE2) human receptor [7,8] and recently it has been demonstrated also for SARS-CoV-2 [9]. Moreover, it has been described that molecular differences in the RBD of SARS-CoV-2 give it potentially higher affinity binding for ACE2, increasing the ability of inter-human transmission [10,11]. Hence, the expression of ACE2 on the cell surface makes it susceptible to SARS-CoV-2 infection. Of note, such cells include type II alveolar cells (AT2) of the lungs [9]. Lung cells are the main target cells despite the fact that ACE2 receptor is widely expressed in non-pulmonary tissues [12,13,14]. The explanation may lie in the extensive expression of the receptor at the level of AT2 cells which therefore could act as reservoirs of infection [15]. In addition, type AT2 cells show high levels of genes required for viral replication [15]. Using single-cell RNA sequencing it has been established that heart, esophagus, kidney, bladder, and ileum are organs at risk for SARS-CoV-2 infection because of significant ACE2 receptor cell expression; according to the results, heart cells were identified as high vulnerable cells, especially during viremia [15].

## 3. Cardiovascular Features of Infected Patients

The epidemiological association between cardiovascular diseases or risk factors and HCoV infection has been previously demonstrated by many studies describing clinical features of SARS and MERS patients [16,17]. In particular hypertension and diabetes were the most prevalent comorbidities for SARS and MERS, and were found to be an independent predictor for mortality and morbidity in SARS patients [16,17].

Since the early published cohort studies, including COVID-19 patients, it emerged that SARS-CoV-2 was more likely to affect older men with cardiovascular comorbidities [18,19,20]. Among the overall COVID-19 Chinese population, 23.7% had at least one coexisting illness [21], mostly chronic disease, such as hypertension, diabetes and coronary artery disease [18,19,20,22,23,24,25]. Two recent meta-analyses on the clinical characteristics of COVID-19 patients reported the overall prevalence of cardiovascular and metabolic diseases [26,27]. In both studies, the most prevalent cardiovascular comorbidities were hypertension (17.1% and 17%), cardio-cerebrovascular disease (16.4% and 5%), and diabetes (9.7% and 8%) [26,27]. Males were more affected than females (57.8% and 51.6%, respectively) [26,27].

An analysis of 5700 patients with COVID-19 admitted to 12 hospitals in New York City, Long Island, and Westchester County from March 1, 2020, and April 4, 2020, showed the most common comorbidities were hypertension (3026; 56.6%), obesity (1737; 41.7%), and diabetes (1808; 33.8%) [28]. Furthermore, 595 patients (11%) had coronary artery disease and 371 (6.9%) congestive heart failure. This study showed that older persons, men, and those with pre-existing hypertension and/or diabetes were highly prevalent and the pattern was similar to data reported from China [28]. A recent Italian multicenter observational study including 196 hospitalized patients confirmed the prevalence of cardiovascular risk (CV) factors in COVID-19 patients; in particular hypertension, diabetes and coronary artery disease were the most prevalent comorbidities [29]. Table 1 summarizes the cardiological clinical features of COVID 19 patients, in Chinese, American (U.S.) and Italian population, compared to those of SARS and MERS patients [17,28,29,30].

In conclusion, based on published studies including those on heterogeneous populations, it is not possible to identify a causal link between CVD and the individual susceptibility to SARS-CoV2 infection; however, the emerging data suggest an epidemiological association with chronic CVD, in particular hypertension, diabetes and coronary artery disease. These clinical conditions should be considered potential risk factors for COVID-19 patients and should be included as red flag in future targeted public health vaccination interventions.

## 4. Myocardial Injury

**Figure 1:**
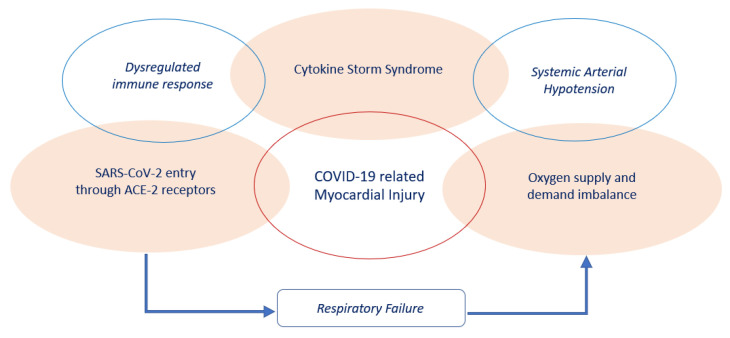
Mechanisms implicated in the pathogenesis of COVID-19 related myocardial injury.

The pathogenesis of acute myocardial injury in the clinical context of COVID-19 remains unclear, however the following three mechanisms may play an important role in its genesis (Figure 1).

First, SARS-CoV-2 may directly cause myocardial damage by entering cardiomyocytes using the ACE2 receptor, which is highly expressed in the heart [30]. Second, myocardial injury may result from the imbalance between oxygen supply and demand due to respiratory failure and systemic arterial hypotension, especially in patients with low ischemic threshold related to underlying CVD [31]. Third, the SARS-CoV-2 infection may lead to dysregulated immune response with higher neutrophil-lymphocyte-ratio (NLR), lower levels of both T helper and T suppressor cells, and higher expression of proinflammatory cytokines (i.e., tumor necrosis factor (TNF)-α, interleukin (IL)-2R and IL-6), chemokines (IL-8), granulocyte-colony stimulating factor, interferon-γ inducible protein 10, monocyte chemoattractant protein 1, macrophage inflammatory protein 1-α [19,32].

This condition, known as “cytokine storm syndrome”, might have a role in cardiovascular system injury, causing direct cardiac toxicity, a rapid onset of severe cardiac dysfunction and a vascular leakage with peripheral and pulmonary edema [33,34,35].

The myocardial zymogram, including the measurement of creatine kinase (CK) and lactate dehydrogenase (LDH) activities, was frequently described in COVID-19 cohort studies [18,21,25].

In a large multicenter retrospective study including 1099 COVID-19 confirmed patients from 552 hospitals in 31 provinces in China, the elevation of CK (≥200 U/L) and LDH (≥250 U/L) serum levels accounted to 13.7% and 41%, respectively [21]. The CK and LDH abnormal serum levels were more highly expressed in critically ill patients (19% and 58.1%, respectively) and in those with major composite endpoint events, including admission to the ICU, invasive mechanical ventilation and death [21].

According to the fourth universal definition of myocardial infarction, myocardial injury is defined by an elevation of cardiac troponin (cTn) value above the 99th percentile URL [31]. The injury is considered acute if there is a rise and/or fall of cTn values [31]. Few studies have described myocardial injuries in COVID-19 patients and its impact on clinical prognosis needs to be clarified.

An early prospective study by Huang et al. [19], including 41 admitted hospital patients with laboratory-confirmed SAS-CoV-2 infection at Jin Yintan Hospital in Wuhan, showed a substantial increase of high sensitive troponin I (hs-cTnI) levels (>28 ng/L) in five patients (12%), in whom the diagnosis of virus-related cardiac injury was made. Four patients were admitted to the ICU, accounting for 31% of the total number of ICU patients [19].

In the retrospective, single-center case series by Wang et al. [23] including 138 consecutive patients with confirmed SAR-CoV-2 pneumonia hospitalized at Zhongnan Hospital of Wuhan University, ten cases (7.2%) were diagnosed with acute myocardial injury. Among them, eight were admitted to the ICU, accounting for 22.2% of the total number of ICU patients [23].

In a retrospective, multicenter cohort study by Zhou et al. [22] including 191 COVID-19 patients from Jinyintan Hospital and Wuhan Pulmonary Hospital, Wuhan, China, 24 patients (17%), out of 145 cases with troponin determinations, showed increased serum levels of cTn I (>28 ng/mL) [22].

In a retrospective analysis by Chen et al. [20] including 150 COVID-19 patients admitted to Tongji Hospital, Tongji Medical College, Huazhong University of Science and Technology, in 22 cases (14.7%) the cTnI value was elevated (>26.3 ng/L); moreover, myocardial injury was present in 62.5% of patients in critical conditions and it seemed to be an independent risk factor (OR = 26.909, 95% CI 4.086 to 177.226, *p* = 0.001) for COVID-19 critical illness patients at multivariate analysis [20].

All these clinical studies, although heterogeneity was considerably high [36], have shown that cTnI serum levels were only marginally increased in COVID-19 patients at hospital admission, observing values exceeding the 99 th percentile in the upper reference limit (URL) in 7–17% of hospital admitted positive cases. Moreover, cTnI values are significantly increased in COVID-19 critically ill patients compared to those with milder forms of disease [36].

These preliminary data suggest the hypothesis that early measurement, at hospital admission of cardiac damage biomarkers in COVID-19 patients, as well as longitudinal monitoring during hospital stay, may help to identify patients at increased risk of evolving toward a worse clinical condition.

## 5. Myocardial Injury Pharmacological Treatment

According to the expert recommendations for clinical management of myocardial injury associated with SARS-Cov-2 infection [37], in addition to close monitoring, symptomatic treatment and respiratory support, treatment with Sodium creatine phosphate (1 g once or twice daily, intravenous infusion within 30 to 45 min); Coenzyme 1 (5 mg once daily, intravenous infusion); Coenzyme Q10) 10 mg three times daily, orally]; Trimetazidine (20 mg three times daily, orally); and Vitamin C (10 g once day intravenous drip, for 15 to 30 days) is recommended to improve myocardial energy metabolism.

In the clinical setting of acute myocardial infarction (AMI) with ST elevation (STEMI) in suspected or diagnosed SARS-CoV-2 infected patients, some Chinese authors [38] suggest performing percutaneous coronary intervention (PCI when hemodynamic instability is present and to prefer thrombolytic therapy in isolated conditions in the case of STEMI stable patient with symptoms onset time within 12h. Out of this temporal range or in AMI patients without ST elevation (NSTEMI) a comprehensive assessment of the risk-benefit ratio of PCI (given limited data on primary PCI benefit for type-2-MI from acute viral illness) and infection control or nosocomial infection should be performed.

A recent guidance from the European Society of Cardiology (ESC) for the diagnosis and management of CV Disease during the COVID-19 pandemic suggests considering all STEMI patients as positive to SARS-CoV-2 infection until laboratory results and to proceed with invasive treatment within 120 min. Fibrinolysis should be performed only in cases where it is not possible to access the catheterization laboratory within two hours after diagnosis. Hemodynamically unstable NSTEMI patients need to be managed as in STEMI, otherwise management should be guided by risk stratification. In case of high risk stable patients, ESC guidance suggests postponing the angiographic study for up to 24 h to wait test results for SARS-CoV-2 infection [39]. In the remaining cases it is always useful to wait for the results of anti-SARS-CoV-2 tests, dismissing alternative diagnoses using non-invasive diagnostic strategies to make risk stratification more accurate and faster in order to decide possibly upon a conservative strategy and rapid hospital discharge [39]. In all cases (STEMI and NSTEMI), testing for SARS-CoV-2 infection should be performed as soon as possible irrespective of treatment of choice and positive patients managed in a COVID-19 hospital.

## 6. Myocarditis

Myocarditis is an inflammatory disease of the myocardium, diagnosed by established histological, immunological and immune-histochemical criteria, caused by infectious, immune-mediated or toxic agents [40]. A previous case report described coronavirus-associated myocarditis in animal models [41], as well as in humans during MERS-CoV infection [42].

To date, sporadic cases of myocarditis have been reported in COVID-19 patients at first clinical presentation of disease [43,44], alone or in the clinical context of pneumonia [45].

In two Chinese cohort studies including 334 and 150 COVID-19 patients respectively, the prevalence of myocarditis ranged from 0.3% to 7% [46,47].

Based on published data, SARS-CoV-2 may represent a potential novel etiology of fulminant myocarditis. It should be suspected in COVID-19 patients with acute-onset chest pain, ST segment changes, cardiac arrhythmias, and hemodynamic instability. In addition, left ventricular dilatation, global/multi-segmental left ventricular hypo-contractility [on echocardiography), and significant increase in cardiac troponin levels, without significant coronary artery disease, could also be present [39].

Actually, even if cardiac injury is not uncommon in COVID-19 patients, myocarditis seems to be a rare event, but when occurring, appears early in patients’ clinical history [48].

## 7. Arrhythmias

There are few and conflicting data about the occurrence of arrhythmias in the context of COVID-19; however, the clinical presentation seems to be not different from those described in the general population (i.e., palpitations, dyspnea, dizziness, chest pain, syncope, etc.) and occurs in 7.3% of COVID-19 cases as the initial symptom of the myocardial involvement [49].

In a cohort study by Chen et al. [20] including 150 COVID-19 patients, arrhythmias were present at admission only in 1.3% of patients. In the cohort study by Wang et al. [23] an arrhythmia complicated the clinical course of the disease during hospitalization in 16.7% of COVID-19 patients; moreover, arrhythmias were significantly higher in patients receiving ICU care than in those not receiving ICU care (44.4% vs. 6.9%; P < 0.001). In both studies, the authors did not describe the type and duration of arrhythmias.

In contrast to these early experiences, the two largest observational studies, including 1099 Chinese and 5700 U.S. COVID-19 patients respectively, did not report any arrhythmias [21,28]. A recent Italian multicenter observational study showed a prevalence of 12.5% among hospitalized COVID-19 patients [29]; moreover, a report by the COVID-19 Task Force of the Italian National Institute of Health showed that 22.5% of non-surviving COVID-19 patients (n: 355, mean age 79.5 years, 30% women) presented atrial fibrillation as comorbidity diagnosed before the SARS-CoV-2 infection [50]. The approach to atrial fibrillation (AF) management should take into consideration several pharmacological interactions between antiarrhythmic and experimental COVID-19 therapies [51]

Ventricular arrhythmias seem to be directly correlated to COVID-19 induced myocardial injury Among a cohort of 187 COVID-19 patients without history of arrhythmias, the subjects with elevated TnT levels more frequently developed malignant arrhythmias, including ventricular tachycardia and fibrillation during hospitalization (2 (1.5%) vs. 9 (17.3%) compared to those with normal TnT levels [52].

Based on these few data, arrhythmias represent a not rare clinical presentation of COVID-19 that could complicate the clinical course of the disease during hospitalization and worsen the prognosis of infected patients, presenting high prevalence in not-survived patients. For this reason, careful electrocardiographic monitoring should be performed in COVID19 patients to early detect paroxysmal arrythmia that does not match the disease status and might be a red flag of worsening disease [53].

## 8. Heart Failure

Heart failure (HF) was reported by Zhou et al. in 23.0% of hospitalized COVID-19 patients and it was more common in patients who did not survive the hospitalization compared to those who did survive (51.9% vs. 11.7%, *p* < 0.001) [22]. The high HF prevalence among COVID-19 patients might be related to failure of pre-existing cardiomyopathy with impaired left ventricular function or it might be consequent to COVID-19 related myocardial injury, both through direct and immune-mediated mechanisms. Right heart failure with increased pulmonary vascular resistance and pulmonary hypertension should also be considered, especially in the context of moderate-severe pneumonia and ARDS [53]. The coexistence of HF and COVID-19 makes diagnosis and management more complicated [54]; however, there are significant differences in chest CT when HF stands alone or is diagnosed simultaneously with COVID-19 such as enlargement of pulmonary veins, lesions distribution and morphology [54]. Based on published data, heart failure might represent a severe complication of COVID-19. The early recognition of HF symptoms and timely HF treatment may be of pivotal importance to improve the prognosis of COVID-19 patients: in fact, when heart failure falls into cardiogenic shock, refractory to therapy, the treatment of these patients becomes rather complicated [55]. In presence of cardiogenic shock, refractory to therapy, the Extracorporeal Life Support Organization [ELSO] guidelines recommend the use of veno-arterial extra corporeal membrane oxygenation (VA-ECMO) as the bridge to recovery [56]. As far as cardiogenic shock complicating COVID-19 is concerned, little evidence is offered for the use of VA-ECMO in these patients [44,57]. Given the lack of documented experience, some working groups have proposed general recommendations for the use of this intensive care treatment, focusing attention on the patient’s age, the presence of sepsis, the coagulation status, and the presence of ARDS and/or MOF, whose presence can frustrate the use of this device [58,59].

## 9. Cardiovascular Outcomes

The evidence emerging from the early Chinese cohort study including COVID-19 patients showed a significantly increased prevalence of cardiovascular diseases (CVD) in critically ill patients compared to those with milder forms of disease [18,23] and, in the same way, in non-surviving COVID-19 patients compared to survivors [46]. In a retrospective analysis by Chen et al. [20] including 150 COVID-19 patients, a history of coronary heart disease was present in 6.7% of patients in critical conditions and this seemed to be an independent risk factor for the need of ICU care at multivariate analysis (OR = 16.609, 95% CI 2.288 ~ 120.577, *p* = 0.005).

Zhou et al. [22] in a retrospective multicenter cohort study including 191 COVID-19 patients (median age 56·0 years IQR 46·0–67·0) from Jinyintan Hospital and Wuhan Pulmonary Hospital found that odds of in-hospital death were higher in COVID-19 patients with coronary heart disease (OR: 21·40; 95% CI 4·64–98·76, P < 0·0001); however, this evidence did not reach statistical significance at multivariate analysis.

A report of the Chinese Center for Disease Control and Prevention [60] showed that the all-cause death incidence rate was 2.3% with an average risk of death of 0.015 per patient observed for 10 days, among 44,672 confirmed cases in Mainland China. The crude case fatality rate was 2.8% for men and 1.7% for women, with the highest percentage (14.8%) among octogenarians. The fatality rate in COVID-19 patients with cardiovascular diseases was higher than in those with no comorbidities (10.5% vs. 0.9%), diabetes (7.3%), chronic respiratory disease (6.3%) and malignancy (5.6%).

The most recent statistics emerging from Italy, which saw one of the largest and most serious clusters of COVID-19 in the world, showed that the case-fatality rate has been very high and is dominated by very old patients. According to the data by the COVID-19 Task Force of the Italian National Institute of Health about 60.7% of COVID-19 related deaths had three or more pre-existing chronic CVDs, 21.2% had two, and 14.4% at least one comorbidity. Hypertension (69.7%), coronary artery disease (27.4%) and diabetes (31.9%) were the most prevalent cardiac comorbidities [61].

Peng et al. [62] from a large cohort of 406 COVID-19 patients admitted to the Western Hospital of Union Hospital in Wuhan performed a retrospective analysis to explore the clinical outcome of 112 COVID-19 patients with CVD (27.6%), in particular hypertension (81.4%), coronary artery disease (55.4%) and heart failure (35.7%). The total incidence of all-cause mortality was 15.18% (17/112); respiratory failure was the leading cause of death (64.7%), followed by AMI (17.6%). Compared to survived COVID-19 patients, non-surviving COVID-19 patients with CVD were more likely to have, or more often had, BMI> 25 kg/m 2 (88.2% vs. 18.95%, *P* < 0.001), coronary heart disease (88.24% vs. 49.47%; *p < 0.03*) and heart failure (76.47% vs. 28.42%; *p < 0.001*) [62].

In conclusion, from the analysis of published data it seems clear that COVID-19 patients with underlying CVD are more likely to develop a severe degree of the disease and death; however, there is currently no evidence that one specific CV risk factor could be per se an independent risk factor for severe complications or death in COVID-19. Based on severe and poor prognosis of COVID-19 patients with underlying CVD, this special group needs preventative and intensive treatment of past cardiovascular disease.

## 10. Renin-Angiotensin-System (RAS) Inhibitors

Previous studies have identified ACE2 as a functional receptor for HCoVs [7,8], including SARS-COV-2 [6]. ACE2 is a human homologue of ACE and degrades Angiotensin II to generate Angiotensin 1-7, thereby negatively regulating the renin-angiotensin system (RAS). Differently from its ubiquitous homologue ACE, ACE2 is abundantly expressed in airway epithelial cells and plays a crucial role in SARS-CoV-induced lung injury [63,64].

All published cohort studies analyzing the clinical features of COVID-19 patients showed hypertension and diabetes as the most prevalent cardiovascular comorbidities [21,26,27]. Based on the experimental data on the increased expression of ACE2 in patients with diabetes [11] and hypertension [65] while on ACE inhibitors (ACE-I) or angiotensin II type-I receptor blockers (ARBs) treatment, some authors [11,65,66,67] hypothesized that their use might increase the risk of SAR-COV-2 infection and develop a severe form of COVID-19, through the upregulation of ACE2 expression. Intravenous infusion of ACE inhibitors and angiotensin receptor blockers (ARBs) in experimental animal models increase the numbers of ACE2 receptors in the cardiopulmonary circulation [68].

Conversely, following the evidence of a reduced expression of ACE2 in the lungs of mice infected by SARS-CoV [63] and based on the attenuation of lung injury by blocking the renin-angiotensin pathway in the human cellular model [69], other authors [70] suggest a protective effect of ACE-I/ARBs from SARS-CoV-2 infection and worse presentation of the disease.

In any case, treatment with an ACEI or ARB may downregulate the expression of ACE2 but it seems not to have significant effect on its activity [71]. In fact, it has been demonstrated that there is a lack of correlation between the rise and fall of cardiac ACE2 mRNA expression and its activity: ACE2 mRNA expression increases with lisinopril isolated treatment, but ACE2 activity did not increase correspondingly, while cardiac ACE2 mRNA expression levels and activity increase after treatment with losartan alone [71].

**Figure 2:**
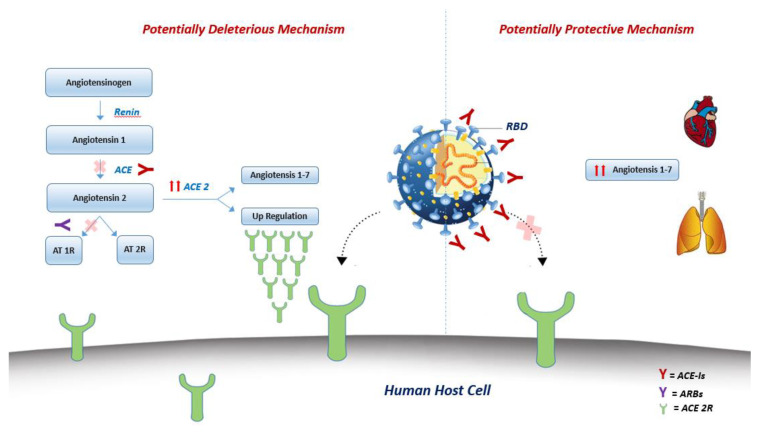
Conceptual figure showing the central role of the treatment with ACE inhibitors/ARBs in the potentially deleterious *[left side picture]* or protective *[right side picture*] effect on lungs in the clinical context of COVID-19. **Potentially deleterious mechanism:** renin-angiotensin-aldosterone system (RAAS) blocking drugs, such as ACE-Is and ARBs, might increase the activity of ACE 2 with consequent upregulation of ACE 2R, responsible of SARS-CoV-2 entry into host cells. **Potentially protective mechanism:** ACE-Is might bind the RBD of SARS-CoV-2 and thereby affect its interaction with ACE2, interfering host cells entry. Moreover, ACE2 might catalyze the conversion of angiotensin II to angiotensin 1–7, which acts as a vasodilator and exerts protective effects in the cardiovascular system. ACE-Is = ACE Inhibitors; ARBs = Angiotensin II type-I receptor blockers; RBD = Receptor-binding domain.

Since ACE-Is bind the catalytic center rather than the receptor-binding domain (RBD) site of the receptor, they might indirectly alter the RBD of SARS-CoV-2 and thereby affect its interaction with ACE2 [10] (Figure 2).

An early experiment by Peng et al. [62], analyzing the impact of antihypertensive therapy on clinical outcome among 112 COVID-19 patients with cardiovascular disease, did not show any significant difference in ACE-I/ARB use percentage whether in COVID-19 critically ill patients compared to those with milder forms of disease, or in not-survived COVID-19 patients compared to survivors. A recent large Chinese study [72], analyzing the use of ACEIs/ARBs among 1178 COVID-19 patients with hypertension and other comorbidities, did not show differences in disease progression and mortality between patients treated with and without ACEIs/ARBs.

These data support the strong recommendations, by the main international cardiovascular scientific societies, to maintain or initiate ACEIs/ARBs treatment in patients with hypertension, heart failure or myocardial infarction, irrespective of SARS-CoV-2 infection, unless there is an alternative clinical reason to suspend them. Actually, the morbidity and mortality risk of stopping such drugs is significant, particularly given the myocardial damage that may occur and the lack of clinical or scientific evidence suggesting the discontinuation of this treatment in COVID-19 patients.

## 11. Pharmacological COVID-19 Experimental Therapy and Cardiovascular Implication

### 11.1. Antiviral Drugs

No specific antiviral drugs are available for SARS-CoV-2 infection. To date, the strategy consists in off-label administration of antiviral drugs effective in other viral infections.

#### 11.1.1. Remdesivir

Remdesivir, an adenosine analogue, showed antiviral properties against SARS-Cov-2 infection and ability to block post-entry viral replication in an in-vitro study [73] and some case studies have reported clinical benefits in critically ill SARS-CoV-2 infected patients [74,75,76] There are several current clinical trials testing Remdesivir effects on COVID-19 patients [77]. On April 2020 Wang et al. published results of a randomized, double-blind, placebo-controlled, multicenter trial conducted to assess the effectiveness and safety of intravenous remdesivir on 237 SARS-CoV-2 patients [73]. No significant differences were found in the remdesivir group compared with placebo in time to clinical improvement but a trend toward a faster recovery was found in those patients with symptoms onset of less than 10 days. No significant differences were also found between groups for 28-day mortality. Adverse events were similar between groups, apparently lower in the remdesivir one; however, because of gastrointestinal symptoms, aminotransferase or bilirubin increases, and worsened cardiopulmonary status, a considerable proportion of patients on remdesivir had to prematurely stop the treatment [73] Other trials are certainly needed to confirm these findings especially on the use of remdesivir in the earlier stage of the disease due to the lack of representation of this subset of patients in the present study.

Generally, direct cardiovascular toxicity or specific medication interactions have yet to be reported for remdesivir. One case was previously described among 175 Ebola patients who developed severe hypotension and subsequent cardiac arrest after remdesivir loading dose [78].

#### 11.1.2. Favipiravir

Favipiravir, a pyrazine carboxamide derivative, is the first approved antiviral drug by the National Medical Products Administration of China for the treatment of COVID-19 (www.nmpa.gov.cn). It has shown efficacy in treating the infection with minor side effects in a Chinese clinical trial including 70 COVID-19 patients (unpublished data). It was previously suggested that Favipiravir administered at high doses may contribute to QT interval prolongation in Ebolavirus infected patients [79]; however, this effect was lacking in healthy Japanese adults [80].

#### 11.1.3. Lopinavir/Ritonavir

Lopinavir/Ritonavir, two protease inhibitors, did not significantly accelerate clinical improvement, reduce mortality, or diminish throat viral RNA detectability compared to standard care in a randomized clinical trial including 119 patients with severe COVID-19. Moreover, the Lopinavir/Ritonavir group showed more adverse events, especially gastrointestinal, leading 13 patients to stop treatment prematurely [81]. Lopinavir/Ritonavir may result in PR and QT interval prolongation and caution should be used in patients with baseline electrocardiographic abnormalities and those taking other QT prolonging drugs [82]. Lopinavir/ritonavir inhibits the activity of CYP3A4 [83] and P glycoprotein [84] and thereby may influence the activity of P2Y12 with implications on non-vitamin K oral anticoagulants and antiplatelet drugs serum concentrations [85] (Table 2).

### 11.2. Chloroquine and Hydroxychloroquine

Chloroquine, an amine acidotropic form of quinine, is used for malaria treatment and prophylaxis [86], has immunomodulant and anti-inflammatory properties [87] and seems to have a broad-spectrum antiviral activity, by increasing endosomal pH required for virus/cell fusion, as well as interfering with the glycosylation of cellular receptors of SARS-CoV [87,88].

Recently, Wang et al, [73] evaluated in vitro five FDA-approved drugs (ribavirin, penciclovir, nitazoxanide, nafamostat, chloroquine) and two broad-spectrum antiviral drugs (remdesivir and favipiravir) in a clinically isolated SARS-CoV-2 patient. The authors concluded that chloroquine was effective and safe in the control of SARS-CoV-2 infection in vitro and suggested its assessment in COVID-19 patients. At least 23 different trials for SARS-CoV-2 already registered in the Chinese Clinical Trial Registry propose to use chloroquine or hydroxychloroquine in the treatment of COVID-19 [89].

Thus far, results from more than 100 patients demonstrated that chloroquine phosphate is superior to the control treatment in inhibiting the exacerbation of pneumonia, improving lung imaging findings, promoting a virus negative conversion, and shortening the disease course. Severe adverse reactions to chloroquine phosphate were not noted in the aforementioned patients [90]. Hydroxychloroquine, the 4-aminoquinoline form of chloroquine, has already demonstrated anti-SARS-CoV activity in vitro [91] and recently it was found to be more potent than chloroquine in inhibiting SARS-CoV-2 infection in an in-vitro study [92]. Currently various protocols are available for chloroquine and hydroxychloroquine administration according to different expert consensus documents [93,94].

Recently the Data Safety Monitoring Board of the Solidarity trial, an international clinical trial launched by the WHO and partners, selecting remdesivir, lopinavir/ritonavir, lopinavir/ritonavir with interferon beta-1a, and hydroxychloroquine as initial treatment options, has investigated data that emerged from a multinational registry analysis showing that the use of hydroxychloroquine or chloroquine with or without a macrolide for treatment of COVID-19 was associated with a decreased in-hospital survival and an increased frequency of ventricular arrhythmias. On June 3 2020 the WHO’s Director-General announced that on the basis of the worldwide available mortality data there are no reasons to modify the Solidarity trial protocol, including the chloroquine and hydroxychloroquine arm, and the study by Mehera et al. was finally retracted [95,96].

In any case, caution is required in establishing indications and contraindications for use of chloroquine in COVID-19 patients, with special mention to the pro-arrhythmic effects of the drug deriving from its ability to lengthen the QT tract [97] and subsequent risk of torsades de pointes [98,99,100]. Moreover bradyarrhythmias have been observed with hydroxychloroquine treatment [98,101,102], potentially leading to heart block with a stepwise manifestation beginning with right bundle branch block, then left anterior fascicular block, and ultimately third degree AV block [103], needing permanent pacemakers [102,104]. Major conduction abnormalities are more common in chronic use than in acute setting [105,106] and more often associated with chloroquine than with hidroxychloroquine treatment [101].

Long-term chloroquine and hydroxycloroquine use is also associated with cardiomyopathy [107,108,109,110,111] often under recognized by healthcare providers [110], leading to preventable adverse events. Although cardiomyopathy phenotype can vary, the typical presentation is that of a non-genetic hypertrophic cardiomyopathy (MOGE(S) classification M_H_ O_H_ G_N_ E_t-CQ_) [112].

### 11.3. Tocilizumab

The cytokine storm seems to be a major determinant of COVID-19 clinical severity [19,33,113,114]. Tocilizumab, an interleukin-6 (IL-6) receptor antagonist, is currently approved by the Food and Drug Administration [FDA] for treatment of rheumatoid arthritis, giant cell arteritis, polyarticular juvenile idiopathic arthritis, systemic juvenile idiopathic arthritis and cytokine release syndrome (http://www.fda.gov). Encouraging but unpublished results have been obtained from off-label use of tocilizumab in COVID-19 patients. In this regard, the Italian Drug Agency (AIFA) has recently approved an ad hoc clinical trial for its use against SARS-CoV-2 infection (https://www.aifa.gov.it). Clinical trials comparing tocilizumab with continuous renal replacement therapy (CRRT) in cytokine release syndrome and combination therapy studies with tocilizumab and antiretroviral (favipiravir) therapy in COVID-19 patients have recently been registered [115,116] The Food and Drug Administration warns that high blood pressure is one of the most common adverse events with tocilizumab [117]. Previous studies showed that tocilizumab was associated with increased mean lipid levels (total cholesterol, high-density lipoprotein (HDL) cholesterol, LDL cholesterol, and triglyceride) [118,119] and interacts with CYP450 which is also implicated in atorvastatin metabolism [120]. These changes tend to occur within the first few weeks of treatment and remain stable during long-term therapy [121]. For this reason, prudent use of tocilizumab has always been made in patients with a high cardiovascular risk profile, with particular attention to dyslipidemic patients. Even so, a recent systematic review and network meta-analysis showed that, despite an increase in cholesterol levels, tocilizumab has safe cardiovascular outcomes compared to other antirheumatic drugs [122].

### 11.4. Convalescent Plasma

Among supportive care, convalescent plasma was found useful in previous SARS pandemics [123,124,125]. Empirical treatment with convalescent plasma had been approved during the Ebola virus outbreak in 2014 and a specific protocol was designed for MERS infection in 2015 [126]. Convalescent plasma reinfusion in H1N1 infection showed lower mortality, especially in the most serious patients, with no side effects [127]. Several case reports and case series have shown clinical benefits and no clinically relevant adverse effects with use of SARS-CoV-2 convalescent plasma reinfusion [128,129], so the treatment has been approved by the Chinese national health committee for critical ill patients [130]. To date there are at least 10 ongoing trials to assess effectiveness and efficacy of convalescent plasma for COVID-19 disease treatment [131].

## 12. Drug interaction

Major drug interactions are shown in Table 3.

## 13. Conclusions

There is a bidirectional relationship between COVID-19 and CVD. If, on the one hand, patients with underlying CVD seem to be at higher risk of contracting COVID-19 and usually present a worse prognosis, on the other hand a not negligible number of infected patients experienced cardiovascular acute events in addition to other COVID-19 related complications. More attention should be paid to patients presenting with myocardial injury, defined by extremely increased cardiac troponin I (cTnI) levels or to new-onset arrhythmias, because these might deteriorate rapidly with ARDS, septic shock, MOF, and death. All physicians involved in COVID-19 management should be aware of the cardiovascular implications of the disease. Moreover, considering the high prevalence and worse prognosis of CVD among this population, we warmly recommend the presence of a cardiologist with extensive experience in intensive care medicine as essential part of the COVID-19 care team (Table 4). One of the main targets of the COVID-19 care team should be to create detailed protocol management of COVID-19 patients with cardiovascular complications and/or COVID-19 with pre-existing CVD, to guarantee the best care for patients and maximum safety for the healthcare providers, nurses and physicians. 

## Figures and Tables

**Table 1:** Comparison between cardiological clinical features of SARS, MERS and COVID-19.

.	*SARS ^(28,30)^*		*MERS ^(17,30)^*		*COVID-19 (Chinese report) ^(27)^*	*COVID-19 (U.S. report) ^(28)^*	*COVID-19 (Italian report) ^(29)^*
**Patients, n**	**357**	**144**	**637**	**245**	**46248**	**5700**	**192**
**Age, mean (range)**	45 (34–57)	45 (34–57)	53 (35–65)	53 (36–66)	56	63 (52–75)	67,7 (52.5–80.9)
**Sex, male n (%)**	157 (44)	70 (49)	426 (67)	154 (63)	23,863 (51,6)	3437 (60.3)	115 (59.9)
**Hypertension, n (%)**	-	-	305 (48)	81 (33)	6474 (17)	3026 (56.6)	111 (57.8)
**Diabetes, n (%)**	21(5,9)	16 (11)	324 (51)	76 (31)	3699 (8)	1808 (33.8)	42 (21.9)
**Cardiovascular Disease, n (%)**	24 (6,7)	17 (12)	197 (31)	37 (15)	2312 (5)	966 (16,9)	26 (13.5)

**Table 2:** Adverse cardiovascular events with COVID-19 experimental therapies.

*Drug*	*Adverse CV events*
*Remdesivir*	One case of severe hypotension and subsequent cardiac arrest after loading dose
*Favipinavir*	Uncertain QT interval prolongation at high doses
*Lopinavir/Ritonavir*	PR and QT interval prolongation
*CQ/HCQ*	QT interval prolongation; Bradyarrhythmias; Cardiomyopathy
*Tocilizumab*	Hypertension; Increased lipid levels

CV: cardiovascular events; CQ/HCQ: chloroquine/hydroxychloroquine.

**Table 3:** Major cardiovascular drug interaction with anti-SARS-CoV-2 drugs.

	LP/RT	CQ/HCQ	TCZ
Acenocumarol	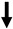	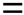	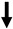
Warfarin	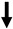	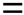	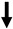
Apixaban *	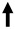	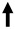	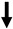
Rivaroxaban	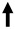	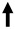	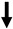
Edoxaban	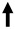	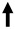	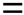
Dabigatran	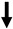	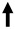	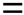
Prasugrel	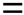	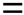	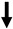
Ticagrerol	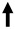	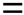	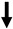
Clopidogrel	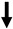	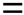	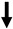
Flecainide	∆ 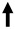	∆ 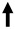	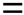
Propafenone	∆ 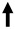	∆ 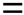	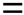
Dofetilide	! 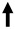	! 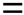	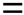
Ibutilide	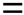	! 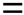	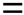
Vernakalant	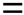	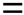	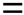
Amiodarone	! 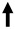	! 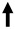	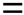
Lidocaine	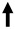	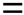	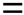
Digoxin	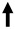	! 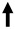	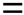
CCB	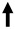	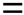	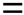
Nicardipine	! 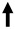	! 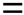	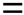
Verapamil	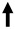	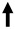	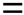
Beta-blockers	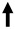	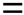	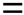
Atorvastatin	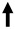	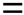	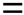
Lovastatin	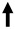	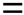	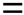
Simvastatin	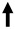	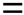	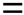

LP/RT:lopinavir/ritonavir; CQ/HCQ: chloroquine/hydroxychloroquine; TZC: tocilizumab; CCB: calcium-channel blockers Red box: avoid interaction; Yellow box: left drug levels needs monitoring; Green box: safe interaction; Arrows/equal symbols indicate what may happen to serum levels of listed drugs; Dashed arrow indicates what happens to anti-SARS-CoV-2 drug; * The US SmPC suggests reduction of dose by 50% in patients taking 5 mg BID and to avoid coadministration in patients already taking apixaban at 2.5 mg BID;∆: possible widening of QRS with increased risk of ventricular arrhythmias; !: QT prolongation. Modified from https://www.covid19-druginteractions.org/.

**Table 4:** COVID-19 and HEART: *HIGHLIGHTS*.

SARS-COv-2 may entry in myocardiocytes causing myocardial injury
Cardiac damage biomarkers may identify COVID-19 patients at increased risk of worse clinical condition or death.
Hypertension, diabetes, and coronary artery disease are the most prevalent comorbidities among COVID-19 patients.
COVID-19 patients with underlying cardiovascular diseases are more likely to develop severe degree of the disease and death.
Fulminant myocarditis is a rare event and appears early in the clinical history of COVID-19 patients.
Arrhythmias represent a not rare clinical presentation of COVID-19 and might complicate the clinical course of disease and worse the prognosis.
The usual cardiovascular therapy, including the anti-hypertensive drugs, should be continued during the SARS-COV-2 pandemic.
There is no scientific evidence to suggest that treatment with ACE-I or ARBs should be discontinued because of the SARS-CoV-2 infection or COVID-19.
All physicians involved in COVID-19 management should be aware of cardiovascular implications of the disease
A cardiologist with high experience in intensive care medicine should be part of COVID-19 care team.
